# Rapid Ultrasound-Assisted Starch Extraction from Sago Pith Waste (SPW) for the Fabrication of Sustainable Bioplastic Film

**DOI:** 10.3390/polym13244398

**Published:** 2021-12-15

**Authors:** Shiou Xuan Tan, Andri Andriyana, Steven Lim, Hwai Chyuan Ong, Yean Ling Pang, Gek Cheng Ngoh

**Affiliations:** 1 Department of Mechanical Engineering, Faculty of Engineering, Universiti Malaya, Kuala Lumpur 50603, Malaysia; kva190014@siswa.um.edu.my (S.X.T.); andri.andriyana@um.edu.my (A.A.); 2 Department of Chemical Engineering, Lee Kong Chian Faculty of Engineering and Science, Universiti Tunku Abdul Rahman, Kajang 43000, Malaysia; pangyl@utar.edu.my; 3 Centre of Photonics and Advanced Materials Research, Universiti Tunku Abdul Rahman, Kajang 43000, Malaysia; 4 Future Technology Research Center, National Yunlin University of Science and Technology, Douliou 64002, Taiwan; onghc@yuntech.edu.tw; 5 Centre of Separation Science and Technology, Department of Chemical Engineering, Faculty of Engineering, Universiti Malaya, Kuala Lumpur 50603, Malaysia

**Keywords:** ultrasound, sago pith waste, sago starch, extraction yield, starch-based bioplastic

## Abstract

The present study was conducted to optimize the extraction yield of starch from sago (*Metroxylon sagu)* pith waste (SPW) with the assistance of ultrasound ensued by the transformation of extracted starch into a higher value-added bioplastic film. Sago starch with extraction yield of 71.4% was successfully obtained using the ultrasound-assisted extraction, with the following conditions: particle size <250 µm, solid loading of 10 wt.%, ultrasonic amplitude of 70% and duty cycle of 83% in 5 min. The rapid ultrasound approach was proven to be more effective than the conventional extraction with 60.9% extraction yield in 30 min. Ultrasound-extracted starch was found to exhibit higher starch purity than the control starch as indicated by the presence of lower protein and ash contents. The starch granules were found to have irregular and disrupted surfaces after ultrasonication. The disrupted starch granules reduced the particle size and increased the swelling power of starch which was beneficial in producing a film-forming solution. The ultrasound-extracted sago starch was subsequently used to prepare a bioplastic film via solution casting method. A brownish bioplastic film with tensile strength of 0.9 ± 0.1 MPa, Young’s modulus of 22 ± 0.8 MPa, elongation at break of 13.6 ± 2.0% and water vapour permeability (WVP) of 1.11 ± 0.1 × 10^−8^ g m^−1^ s^−1^ Pa^−1^ was obtained, suggesting its feasibility as bioplastic material. These findings provide a means of utilization for SPW which is in line with the contemporary trend towards greener and sustainable products and processes.

## 1. Introduction

Generally, starch extraction methods can be categorized into chemical and physical techniques. Acid hydrolysis [1,2] and enzymatic hydrolysis [3] are several notable chemical extraction techniques. Acid hydrolysis requires long processing duration of more than 5 days and extra purification steps [4,5,6]. Meanwhile, enzymatic hydrolysis needs to undergo a lengthy incubation period of more than 24 h to remove protein and fiber prior to the release of starch. Enzymatic hydrolysis is also costly as it requires a pure enzyme and antimicrobial agent to reduce the risk of starch fermentation or degradation [7,8]. Recently, physical techniques such as stirred media milling [9] and high-pressure homogenization [10] had emerged but had constraints such as difficulty in maintaining constant flow at high concentration of starch slurry during milling and the restriction of low concentration starch slurry application, respectively [5]. Comparatively, ultrasound is a better physical technique for starch extraction in providing strong agitation without any external heating source [11,12]. High extraction yield which can be achieved by the simple and fast ultrasound technique without additional chemical treatment outweighs the shortcomings of the conventional chemical and physical techniques [13]. Therefore, ultrasound is favoured for recovering starch from crops such as taro [8], yam [14], corn [6], pea [15], jicama roots [16] and breadfruit [4].

Similar to the starchy crops mentioned above, the residual starch present in the sago (*Metroxylon sagu)* pith waste (SPW) can be extracted and further converted into higher value-added product. SPW represents an abundant source of agro-industrial waste which is derived from sago starch processing with starch content ranges from 60–80% [17]. Approximately one tonne of SPW is generated for every tonne of sago starch produced (dry weight basis). The total amount of SPW produced in Malaysia had been recorded up to 110 tonnes/year which represented a sizeable amount [18,19]. In the conventional sago starch extraction process, low extraction yield (25–41%) was often reported due to the starch granules that rigidly embedded in the fibrous matrices of the sago pith residue [20,21]. Therefore, SPWs are often dumped into rivers and waterways which threatens the aquatic life [17].

As a matter of fact, the starch extracted from the SPW can be plasticized to produce bioplastics. Development of bioplastics is inspired with the aim to reduce the environmental impacts brought by the utilization of petroleum-based plastics [22,23]. Disposal of petroleum-based plastics threatens the environment with extremely long degradation rate which can last for 100–1000 years [24,25]. Furthermore, the emissions of carbon dioxide and other hazardous gases from the incineration of plastic wastes are also detrimental to the environment and human health. Since bioplastics are produced from renewable agricultural resources and biomass feedstock, they could be degraded naturally as carbon dioxide, water and organic matter which are non-toxic to both the environment and humans [24,26,27].

Considering there is no published report on the exploration of SPW as feedstock for starch extraction using ultrasound to the best knowledge, this study aims to evaluate the effect of various parameters (particle size, solid loading, sonication duration, ultrasonic amplitude and duty cycle) towards the extraction yield of starch from SPW. The investigations of these influencing factors are essential to understand their relationship in obtaining the optimum extraction yield within the shortest duration and minimal use of renewable resources. The effectiveness of the ultrasound technique was subsequently compared with the conventional method in terms of extraction yield, extraction efficiency and chemical composition. The granule structure of the starch extracted from both methods was characterized in detailed by scanning electron microscopy (SEM), particle size distribution, X-ray diffraction (XRD), solubility and swelling power. Subsequently, the ultrasound-extracted starch was used to fabricate bioplastic film to validate its feasibility to be used as value-added product. The film properties were also compared with the bioplastic film fabricated using conventional method extracted starch to gauge its effectiveness.

## 2. Materials and Methods

### 2.1. Materials

SPW was provided by Craun Research Sdn. Bhd., Kuching, Malaysia. Potato amylose (100% purity) was purchased from Sigma-Aldrich Co. (St. Louis, MO, USA). Ethanol (95% purity) from HmbG (Selangor, Malaysia). Sodium hydroxide (99% purity), iodine (≥99.8%), potassium iodide (99.5% purity) and acetic acid (100% purity) were obtained from Merck (Darmstadt, Germany). All the chemicals were used as received without further purification. Laboratory formulated distilled water was used throughout the experiments.

### 2.2. Ultrasound-Assisted Starch Extraction from SPW

Starch extraction from SPW was performed following the method of Pinyo et al. [20] with slight modification. Prior to the extraction process, SPW was dried in an oven (SOV70B, Thermo-line, Beijing, China) at 60 °C for 24 h and ground using a blender (Tefal La Moulinette, 1000 W). The SPW powder was then sieved into different particle sizes (smaller than 250 µm, 250–500 µm, 500 µm–1 mm and larger than 1 mm). Starch slurry was prepared by mixing the SPW with distilled water at various solid loadings (5–10 wt.%) and adjusted to a total weight of 200 g. The tip was submerged until half depth of the starch slurry (approximately 1.5 cm to the bottom of the flask). The starch slurry was then subjected to a 500 W one-inch-diameter probe-type ultrasonic processor (model Q500-20, Qsonica LLC, CT, USA) operating at a frequency of 20 kHz. The ultrasound-assisted extraction process was conducted at an auto-induced temperature without temperature control at various amplitudes (50, 60 and 70%), sonication durations (5, 10 and 15 min) and duty cycle (50–100%). The continuous mode (5 s on/0 s off) and 5 different pulse modes (5 s on/1 s off, 5 s on/2 s off, 5 s on/3 s off, 5 s on/4 s off and 5 s on/5 s off) of ultrasonication corresponded to the duty cycle of 100, 83, 71, 63, 56 and 50%, respectively. Upon completion of the ultrasonication, the starch slurry was filtered through a fine filter cloth and the filtrate was allowed to stand for 1 h to precipitate the starch. The supernatant was decanted, and the starch precipitate was dried in an oven at 60 °C for 24 h. Single factor experiment design was adopted to quantify the effects of different factors (particle size, solid loading, ultrasonic amplitude, sonication duration and duty cycle) on the extraction yield of sago starch. The sequence of the factors was decided based on their significance towards the extraction yield found in the literature [28] and the preliminary investigation conducted while minimizing the number of resources required. For control sample, starch was extracted using conventional magnetic stirring approach. Starch slurry was stirred on a magnetic stirrer at 300 rpm and 46 °C for 30 min. The reaction temperature was determined from the reaction conditions for optimum extraction yield using ultrasound technique.

### 2.3. Chemical Composition of Extracted Starch

#### 2.3.1. Determination of Amylose Content

Amylose content of starch was determined according to the iodine colorimetric method of Babu and Parimalavalli [29]. A beaker containing the mixture of 0.1 g of starch sample, 1 mL of 95% ethanol and 9 mL of 1 N sodium hydroxide was heated in a boiling water bath for 10 min with occasional mixing to gelatinize the starch. The cooled solution was then topped up with distilled water to a final volume of 100 mL and was shaken thoroughly. A 5 mL of the solution was taken and placed in another 100 mL volumetric flask covered with aluminium foil. One mL of 1 N acetic acid solution and 2 mL of 0.2% iodine solution were added to the flask and made up to the mark with distilled water. The absorbance of the solution was measured using a UV-vis spectrophotometer (SPEKOL 1500, Analytik Jena, Jena, Germany) at 620 nm. The standard curve was plotted using standards of amylose, with absorbance plotted against known amylose concentration. Amylose content was calculated according to Equation (1).

(1) $$\mathrm{Amylose}\;\mathrm{content}\;\left(\%\right)=\frac{C\times \mathrm{DF}\times V}{W}\times 100\%$$

where:

C is the sample concentration determined from the standard curve (mg/mL);

DF is the dilution factor;

V is the volume of the sample (mL);

W is the weight of the sample (mg).

#### 2.3.2. Determination of Starch Content

Starch content was determined using the enzymatic colorimetric method according to AOAC 920.40 [30]. A measure of 100 mg of the starch sample was incubated with termamyl for 15 min at 100 °C and digested with amyloglucosidase at 60 °C for 30 min. The free glucose content was measured using the spectrophotometer at 510 nm with the aid of the combined glucose oxidase/peroxidase reagent kit. The starch content was calculated by multiplying the free glucose content with a factor of 0.9 [31].

#### 2.3.3. Protein Content

Kjeldahl method was adopted to determine the nitrogen content of the sample which involved digestion, distillation and titration steps. One gram of the sample was digested with 30 mL of concentrated sulphuric acid in the presence of 2.0 g of catalyst mixture (potassium sulfate and selenium). The digested solution was subjected to acidic distillation in the presence of 25 mL of boric acid and 25 mL of distilled water. The distillate was neutralized with sodium hydroxide solution before titrating with sulphuric acid. The protein content was determined by multiplying the calculated nitrogen content with a factor of 6.25 [32].

#### 2.3.4. Determination of Fat Content

One gram of the dried sample was extracted and refluxed in a Soxhlet extractor with *n*-hexane as the extraction solvent for 6 h. The solvent was evaporated using a rotary vacuum evaporator after completion of the extraction. The fat content was determined from the weight of oil retained in the flask [32].

#### 2.3.5. Determination of Ash Content

Two grams of the sample was transferred to a pre-ashed crucible and was combusted over a low burner flame until no more flame was produced. It was then kept in a muffle furnace for overnight at 550 °C. The ash content was determined from the weight of the ash left in the crucible [32].

### 2.4. Determination of Extraction Yield and Extraction Efficiency

Extraction yield was obtained by calculating the amount of dried starch recovered from SPW as expressed in Equation (2) [33].

(2) $$\mathrm{Extraction}\;\mathrm{yield}\;\left(\%\right)=\frac{\mathrm{Weight}\;\mathrm{of}\;\mathrm{dried}\;\mathrm{starch}\;\mathrm{extracted}\;\mathrm{from}\;\mathrm{SPW}\;\left(g\right)}{\mathrm{Weight}\;\mathrm{of}\;\mathrm{SPW}\;\left(g\right)}\times 100\%$$

After determined the optimum conditions, the weight of extracted pure starch was calculated by multiplying the starch content percentage with the weight of dried starch extracted from SPW. Extraction efficiency was calculated according to Equation (3) [33].

(3) $$\mathrm{Extraction}\;\mathrm{efficiency}\;\left(\%\right)=\frac{\mathrm{Weight}\;\mathrm{of}\;\mathrm{extracted}\;\mathrm{pure}\;\mathrm{starch}\;\left(g\right)}{\mathrm{Weight}\;\mathrm{of}\;\mathrm{total}\;\mathrm{starch}\;\mathrm{present}\;\mathrm{in}\;\mathrm{the}\;\mathrm{SPW}\;\left(g\right)}\times 100\%$$

### 2.5. Preparation of Sago Starch Bioplastic Film

The sago starch bioplastic film was prepared following the method of Agustin et al. [27] with slight modification. The starch solution was prepared by mixing 5.0 g of sago starch with 100 mL of distilled water. It was homogenized at 250 rpm and heated at 60 °C for 15 min. A measure of 40 wt.% glycerol (with respect to dried starch) [26] was added into the starch solution and heated at process temperature of 70 °C until it was gelatinized. Upon completion of the reaction, 15.0 g of starch mixture was poured into a square container with dimension of 10 × 10 cm. The mixture was then dried in a heating oven at 60 °C for 24 h and a bioplastic film with thickness of 0.11 ± 0.01 mm was obtained. The film was peeled off and stored in a desiccator with a sealed plastic bag prior to tensile test.

### 2.6. Characterization of Extracted Starch

#### 2.6.1. Scanning Electron Microscopy (SEM)

The surface morphology of the control and ultrasound-extracted starch samples was observed using a bench top scanning electron microscope (Phenom ProX, Thermo Fisher Scientific, Breda, The Netherlands). The samples were dried prior to imaging. A single layer of the starch samples was sprinkled evenly on a clean stub attached with double-sided tape without gold coating. The stubs were then placed in the SEM chamber and the images were captured at an accelerating voltage of 15 kV with magnification of 500× [34]. The average size of the extracted starches was measured on 100 particles randomly using Image J software (National Institutes of Health, Bethesda, MD, USA).

#### 2.6.2. Particle Size Distribution

Particle size distribution and its cumulative curves of both control and ultrasound-extracted starch samples were determined using a laser particle size analyzer (Malvern Mastersizer MSS, Malvern Instruments, Worcestershire, UK) equipped with a HydroMU sample dispersion unit (Malvern Instruments, Worcestershire, UK) at 25 °C [35]. Prior to the analysis, the particle size analyzer and the laser were powered to warm up for 1 h. Beaker containing distilled water was then pumped through the cell twice using the HydroMU sample dispersion unit to remove impurities from the cell. For sample analysis, the amount of sample added into the beaker containing distilled water was in accordance with the obscuration range required by the laser beam of 10–20%.

#### 2.6.3. X-ray Diffraction (XRD) Pattern

The diffraction patterns of the samples were recorded using an X-ray diffractometer (model MiniFlex 300/600, Rigaku, TX, USA). The diffractometer is equipped with a CuKα radiation operating at 45 kV and 30 mA. The diffracted intensity was measured from 3–60° as a function of Bragg angles (2θ) with the scanning speed of 1°/second. Starch crystallinity was calculated according to Equation (4) [16].

(4) $$\mathrm{Crystallinity}=\frac{A_{c}}{A_{c}+A_{a}}\times 100\%$$

where:

$A_{c}$ is the crystalline area;

$A_{a}$ is the amorphous area.

#### 2.6.4. Solubility and Swelling Power

Solubility and swelling power of the control and ultrasound-extracted starch samples were determined according to the method of Sit et al. [8] with slight modification. Starch of 0.5 g (W_0_) and 20 mL distilled water were added into a 50 mL centrifuge tube. The tube was kept in a shaking water bath at 70 °C for 30 min. The suspension was then cooled to room temperature and centrifuged at 2000 rpm for 15 min. The supernatant was carefully decanted in a petri dish and dried in an oven to a constant weight (W_1_) at 103 °C for 2 h. The weight of the swollen granules (W_2_) was measured after decantation. The solubility and swelling power were calculated according to Equations (5) and (6), respectively.

(5) $$\mathrm{Solubility}\;\left(\%\right)=\frac{W_{1}}{W_{0}}\times 100\%$$

(6) $$\mathrm{Swelling}\;\mathrm{power}\;\left(g\;\mathrm{gel}/g\;\mathrm{starch}\right)=\frac{W_{2}}{W_{0}-W_{1}}$$

### 2.7. Characterization of Fabricated Bioplastic Films

#### 2.7.1. Colour Properties

The colour of the bioplastic film was determined using colour spectrophotometer (model CM-5, Konica Minolta, Tokyo, Japan) interfaced with computer operating Spectra Magic NX software (Konica Minolta, Tokyo, Japan). A white standard colour plate was used as the background for the colour measurements. The films were subjected to CIE-Lab system under illuminant D65 and observer 10° where L, a and b represent lightness, redness and yellowness. The total colour difference (ΔE) was calculated using Equation (7) [36]. The average values of the three measurements taken at three different positions of each film were reported.

(7) $$\Delta E=\sqrt{{\left(L\;-{\;L}^{*}\right)}^{2}+{\left(a\;-{\;a}^{*}\right)}^{2}+{\left(b\;-{\;b}^{*}\right)}^{2}}$$

where L*, a* and b* represent the respective values of the white standard colour plate while L, a and b represent the respective values of the film.

#### 2.7.2. Tensile Test

Mechanical properties of bioplastic film such as tensile strength, Young’s Modulus and elongation at break were determined according to ASTM D882-02 [37] using Universal Testing Machine (Autograph AG-X, Shimadzu, Kyoto, Japan) interfaced with computer operating Trapezium software (Shimadzu, Kyoto, Japan). Measurements were performed with load cell of 500 N, crosshead speed of 5 mm/min and grip separation of 30 mm. Three readings were taken from three random places of each film, measuring 7 × 1 cm. The average values of the three measurements were reported.

#### 2.7.3. Water Vapour Permeability (WVP)

A bottle containing 2.0 g of fully dried calcium chloride (0% relative humidity) was sealed on top (11.5 mm in diameter) with the bioplastic film. It was then placed in a desiccator at room temperature of 25 °C with saturated sodium chloride solution (75% relative humidity). The changes in the weight of calcium chloride were recorded for every 1 h interval. The equilibrium state of saturation was reached after 24 h. The WVP of the film was calculated using Equation (8) [38]. Three replicates of each film were performed.

(8) $$\mathrm{WVP}\;\left({g\;m}^{-1}s^{-1}{\mathrm{Pa}}^{-1}\right)=\frac{w\times d}{A\times t\times P\times \left({\mathrm{RH}}_{1}-{\mathrm{RH}}_{2}\right)}$$

where:

w is the weight gained by calcium chloride (g);

d is the average thickness of the film (m);

A is the area of the film exposed for water vapour permeation (m^2^);

t is the time (s);

P is the saturation vapour pressure of water at 25 °C (Pa);

${\mathrm{RH}}_{1}$ is the relative humidity in the desiccator;

${\mathrm{RH}}_{2}$ is the relative humidity inside the bottle.

### 2.8. Statistical Analysis

The experiments were performed in triplicates and all the statistical analysis results were expressed as mean ± standard deviation. For results displayed on graph, standard deviation was indicated by an error bar. Significant differences between the mean values (*p* < 0.05) were accessed by one-way analysis of variance (ANOVA) with Tukey test using OriginPro 2018 software (OriginLab, Northampton, MA, USA).

## 3. Results and Discussion

### 3.1. Effect of Particle Size

**Figure 1:**
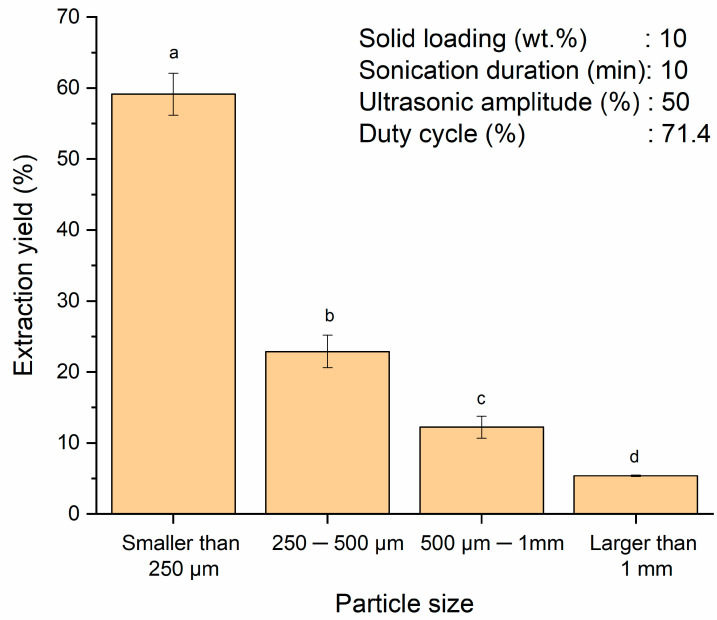
Effect of various particle sizes of SPW (smaller than 250 µm, 250–500 µm, 500 µm–1 mm and larger than 1 mm) on extraction yield of starch from SPW. Different letters indicate the values are significantly different (*p* < 0.05).

Particle size plays an important role in the ultrasound-assisted starch extraction from SPW. Reducing the particle size of the starch increases the number of cells directly exposed to the extraction solvent and the ultrasound cavitation, thus enhancing the extraction yield. This effect could be achieved by grinding the biomass before extraction [39]. Energy consumption increases at lower particle size when the size of the sieve opening changes from coarse to fine [40,41]. Nonetheless, ultrasound could also synergize in breaking down the biomass and prevent agglomeration [39]. Therefore, the smallest particle size adopted in this study was smaller than 250 µm with the purpose of reducing the grinding time and energy. Lower particle size also contributes to higher mass loss and is not economically competitive. Effects of particle size (smaller than 250 µm, 250–500 µm, 500 µm–1 mm and larger than 1 mm) on the starch extraction yield was investigated at a fixed condition (solid loading of 10 wt.%, sonication duration of 10 min, ultrasonic amplitude of 50% and duty cycle of 71.4%). From Figure 1, it can be observed that the starch extraction yield decreased significantly with increasing particle size (*p* < 0.05). The highest extraction yield of 59.1 ± 3.0% was achieved at the smallest particle size of smaller than 250 µm. Shorter diffusion path and larger surface area possessed by smaller particle size would facilitate the mass transfer of the starch, thereby achieving higher extraction yield. Furthermore, reduction in the particle size would lead to an increase in the number of starch cells directly exposed to the solvent, thus improving the extraction yield [42,43]. On the other hand, higher mass transfer limitation associated with larger particle size had hampered solvent penetration for starch release as the starch granules were being trapped between the fibrous material structure of sago [17]. The results showed a significant change (*p* < 0.05) in the extraction yield with the increasing particle size from smaller than 250 µm to larger than 1 mm. Particle size of more than 1 mm recorded the lowest extraction yield at 5.4 ± 0.1%. A similar trend was observed by Shirsath et al. [43] in the curcumin extraction from *Curcuma amada* using ultrasound by varying four different particle sizes (0.09, 0.106, 0.21 and 0.85 mm). The highest (72%) and lowest (55%) extraction yields were achieved at the smallest (0.09 mm) and biggest (0.85 mm) particle sizes, respectively. In the present study, the weight distribution of SPW increased in the descending order of particle size. By grinding the SPW for 30 s and sieving them into different particle sizes, the weight distribution was as follows: 87.7% (smaller than 250 µm), 6.8% (250–500 µm), 4.6% (500 µm–1 mm) and 0.8% (>1 mm). Considering the extraction yield and the weight distribution for particle size smaller than 250 µm were the highest while the weight loss was not very significant which was only approximately 12%, particle size of smaller than 250 µm was selected for the subsequent parameter optimization.

### 3.2. Effect of Solid Loading

**Figure 2:**
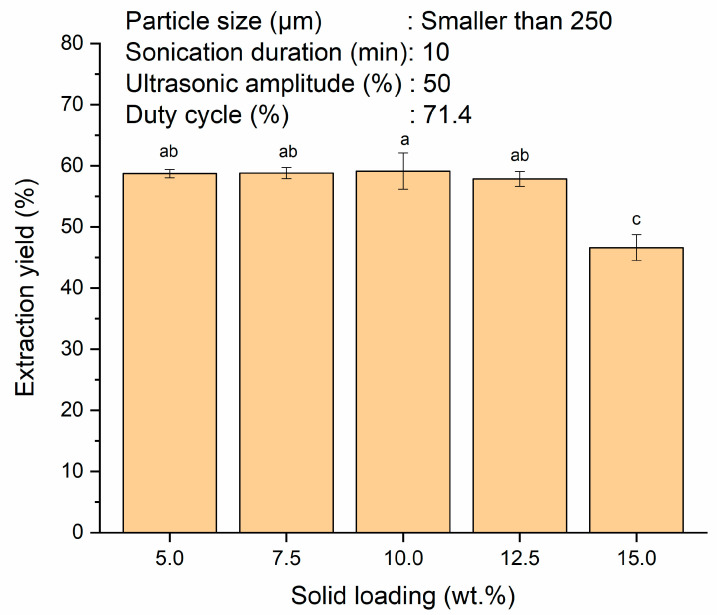
Effect of various solid loadings of SPW (5, 7.5, 10, 12.5 and 15 wt.%) on extraction yield of starch from SPW. Different letters indicate the values are significantly different (*p* < 0.05).

The effect of solid loading on extraction yield was investigated over the range of 5–15 wt.% as depicted in Figure 2. An increase in the solid loading from 5–10 wt.% was able to maintain the extraction yield at 58.7 ± 0.7% and 59.1 ± 3.0%, respectively with insignificant difference (*p* > 0.05). Theoretically, particle collision frequency would increase with higher solid loading with the aid of ultrasound [44]. This enabled the sonication system to cope with larger throughput at higher solid loading. Consequently, the effect of solid loading on the extraction yield was nominal in the present study. This could also be ascribed to the incomplete interaction of the solvent with the solid when the solvent slowly became saturated with increasing solid loading [45]. The extraction yield decreased to 46.6 ± 2.1% when the solid loading was increased to 15 wt.% (*p* < 0.05). This can be explained by the saturation of the solvent beyond the optimum solid loading. The decreasing concentration gradient between the interior starch and the external solvent would lower the mass transfer rate from the solid matrix to the solvent and thus hindered the extraction yield. In addition, different solid loadings will also affect the concentration difference between the inner cells of sago starch and the solvent (water) which subsequently resulting in different viscosities of starch slurry [43]. Increase in the solid loading would increase the viscosity of the starch slurry and caused the ultrasonic energy delivered to the suspension to be partially attenuated. This would lead to a lower effective ultrasonic energy to disrupt the SPW particles in releasing the starch granules and resulted in lower extraction yield [20,44,46]. This phenomenon was also observed by Patil et al. [47] in the ultrasound-assisted extraction of curcuminoids from *Curcuma longa*. The extraction yield reduced from 86.7–31.3% when the solid loading was increased from 5–20% (*w/v)*. At a solid loading of 12.5 wt.%, the amount of starch extracted was 1.2 times higher than that of 10 wt.% solid loading despite its extraction yield was slightly lower (57.9 ± 1.2% vs. 59.1 ± 3.0%). However, more water washing during slurry filtration and longer filtration time were required at solid loading of 12.5 wt.%. Similar extraction yields were also obtained at solid loadings of 5 and 10 wt.%. From the economical point of view, a solid loading of 10 wt.% was chosen for the subsequent parameter study since the final amount of starch produced was doubled compared to the 5 wt.% solid loading.

### 3.3. Effect of Sonication Duration and Ultrasonic Amplitude

**Figure 3:**
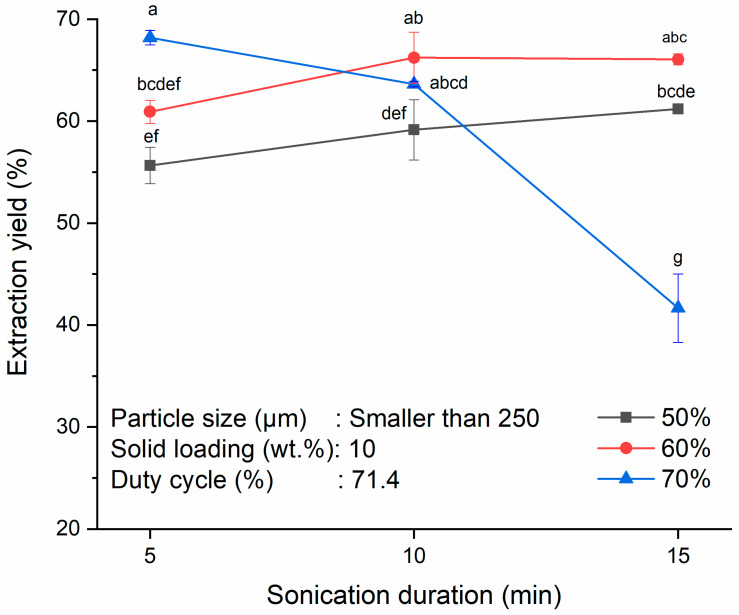
Effects of sonication durations (5, 10 and 15 min) and ultrasonic amplitudes (50, 60 and 70%) on extraction yield of starch from SPW. Different letters indicate the values are significantly different (*p* < 0.05).

Changes in the extraction yield of starch from the SPW at different sonication durations and ultrasonic amplitudes were investigated on particle size of less than 250 µm, solid loading of 10 wt.% and duty cycle of 71.4%. Increment of the sonication duration had enhanced the extraction yield at constant ultrasonic amplitude as shown in Figure 3. When the sonication duration was increased from 5–15 min, the extraction yields increased in tandem from 55.7 ± 1.8% to 61.2 ± 0.3% (*p* > 0.05) and from 60.9 ± 1.1% to 66.1 ± 0.5% (*p* > 0.05) for ultrasonic amplitudes of 50 and 60%, respectively. These results could be related to the dominating effects of both cavitation and thermal effects with increment in sonication duration. Both the cavitation effect and thermal effect are crucial in ultrasound-assisted extraction. The cavitation effect will aid in the imploding of cavitation bubbles while thermal effect will help in the swelling and loosening of the cell structures. Their synergistic effects contribute to the augmented mass transfer of intracellular substances into the solvent [45]. The cavitation effect is associated with more particles breaking down at longer sonication durations which exposes the fibrous material structure of sago to release more starch granules to exterior solvent [20,45,48]. In view of the inherent thermal effect, longer sonication duration would also result in a higher temperature (43–58 °C and 45–63 °C for 50 and 60% ultrasonic amplitudes with sonication duration from 5–15 min, respectively) and a disrupted cell matrix, thus facilitating the starch diffusion from the inner parts of sago to the exterior solvent and enhanced the extraction yield [43,49]. Nonetheless, the increase in the extraction yield with longer sonication duration was not statistically significant (*p* > 0.05) at ultrasonic amplitudes of 50 and 60%.

Conversely, prolonged sonication duration could undermine the extraction yield at high ultrasonic amplitude. This was reflected by the significant decrement in extraction yield at the ultrasonic amplitude of 70% when the sonication duration was increased from 5–15 min (*p* < 0.05). The highest extraction yield of 68.2 ± 0.7% was attained at an ultrasonic amplitude of 70% with sonication duration of 5 min. When the sonication duration was further increased to 15 min, the extraction yield dropped significantly to 41.7 ± 3.3% (*p* < 0.05). The decrease in the extraction yield could be related to the negative thermal effect with prolong sonication duration. Heat generated from the frequent asymmetric collapse of cavitation bubbles could increase the temperature to 66 °C at an ultrasonic amplitude of 70% with sonication time of 15 min. This temperature had exceeded the starch gelatinization temperature of 64 °C, causing some starch in the suspension started to gelatinize. As a result, some starch granules were trapped in the high viscosity suspension after extraction and could not be separated easily [8,16,20,50]. A similar observation was made by Vasudeo [51] in the ultrasound-assisted extraction of starch from cassava in which the gelatinization of cassava starch took place above 50 °C which negatively affected the extraction yield.

On the other hand, extraction yield increased with an increase in the ultrasonic amplitude at constant ultrasonic duration. This was because more energy could be transmitted for the cavitation event to occur and thus, cavitation bubbles generated at higher ultrasonic amplitude could collapse at higher intensity, thereby enabling more cracks and fissures to be formed on the starch surface. Impingement by high-speed jets also caused surface erosion and particle fragmentation simultaneously, leading to more channels and spaces for solvent diffusion. When solvent diffused into the particles easily, it facilitated the starch to be released into the solvent [42,43,52]. Nonetheless, extraction yield obtained at an ultrasonic amplitude of 70% after subjected to 15 min of sonication was lower than that at ultrasonic amplitudes of 50% (*p* < 0.05) and 60% (*p* < 0.05) which can be explained by the aforementioned cavitation and thermal effects. Sit et al. [8] also reported the negative effect of increasing ultrasonic amplitude on taro starch extraction yield due to the degradation and solubilization of the starch. Based on the results obtained in the present study, combination of sonication duration of 5 min and ultrasonic amplitude of 70% gave the highest extraction yield of 68.2 ± 0.7% without the occurrence of starch degradation or gelatinization.

### 3.4. Effect of Duty Cycle

**Figure 4:**
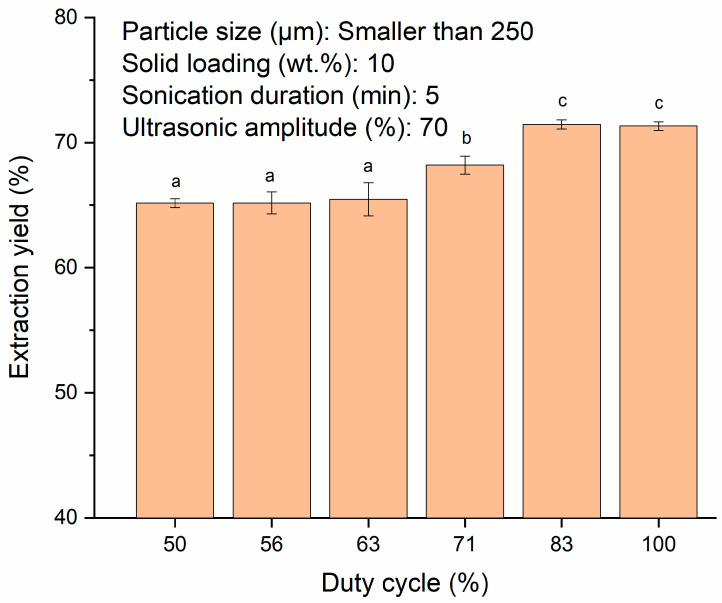
Effect of duty cycles (50, 56, 63, 71, 83 and 100%) on extraction yield of starch from SPW. Different letters indicate the values are significantly different (*p* < 0.05).

Duty cycle is referred as the percentage of ultrasound effective working time to the total ultrasound working time and idling time [53]. It determines the intensity of cavitation effects and prevents excessive burden on the ultrasonicator [54]. Figure 4 shows that as the duty cycle was increased from 50–83%, the extraction yield increased from 65.1 ± 0.4% to 71.4 ± 0.4% (*p* < 0.05). Pulse inflection of the ultrasound suppressed the formation of degassing bubbles and aided in the clearance of the cavitation area and intensified the sonochemical reactions [55]. Higher duty cycle promoted more intensive cavitation activities and elevated the extraction yield [56]. However, there was no significant (*p* > 0.05) difference in the extraction yield between duty cycles of 83–100%. It had been observed that similar extraction yield of around 71% was achieved for duty cycles of 83 and 100% in pulse and continuous mode, respectively. This might be due to the volumetric oscillation in continuous mode which had restricted the cavitation formation. The transmission of ultrasound might be curtailed by the cloud of degassing bubbles through absorption and scattering of the sound waves. The bubbles would then undergo intensive compression and expansion and restrict the sonochemical reaction [55,57]. The same trend was also reported by Dey and Rathod [45] as well as Pan et al. [58] where similar yields were obtained at both pulse and continuous modes in the ultrasound-assisted extraction of β-carotene from *Spirulina platensis* and antioxidants from pomegranate peel, respectively. Continuous mode usually requires shorter sonication time than pulse mode. However, it could accelerate tip erosion and would not be as energy efficient as the pulse mode [58]. Therefore, duty cycle of 83% was considered as the optimum in the present study.

### 3.5. Comparison between Conventional and Ultrasound-Assisted Extraction

To clearly demonstrate the benefits of the ultrasound technique over the conventional method, their extraction yields were compared under the same optimized conditions of particle size smaller than 250 µm, solid loading of 10 wt.% and temperature of 46 °C (temperature auto-generated at ultrasonic amplitude of 70% with duty cycle of 83%). The extraction efficiency at 89.7% was determined based on the total starch present in the SPW. From Table 1, ultrasound techniques could achieve higher extraction yield and efficiency than the conventional approach i.e., (71.4 ± 0.4% vs. 60.9 ± 1.2%) and (89.7 ± 0.5% vs. 77.8 ± 1.5%), respectively. Moreover, the reaction time could be shortened by as much as 83.3% (5 min vs. 30 min) with the assistance of ultrasound in starch extraction. This could be attributed to the larger interfacial area offered by the shock waves generated from the rapid formation of cavitation bubbles in facilitating the starch diffusion [52]. On the other hand, desirable mixing to achieve higher uniformity was difficult for magnetic stirring as the stirring bar was situated at the bottom of the flask which might have resulted in lower extraction yield [59]. Similar findings in the starch extraction from *Radix Puerariae* was reported by Li et al. [60] with an extraction yield of 45.7% achieved by ultrasound technique in 30 min and an extraction yield of 39.6% by conventional approach using magnetic stirrer in 2 h. Comparatively, the ultrasound technique is more energy and time efficient.

Contrary to the lower extraction yield and efficiency, the chemical composition of the extracted starch from the conventional approach recorded higher amylose content than the ultrasound technique (37.4 ± 0.0% vs. 32.1 ± 0.0%). The longer extraction time (30 min) required in the conventional approach may cause more starch granules to swell and thus more amylose would be leached out [61]. In addition, a small discrepancy in the starch content was observed between the conventional and the ultrasound techniques, i.e., 89.3 ± 0.1% vs. 87.8 ± 0.1%. In the starch extraction from *Radix Puerariae* conducted by Li et al. [60], the starch content reported from the ultrasound approach (99.75%) and that from the conventional approach of 99.52% was almost identical too. Lower starch content of the present study could be due to higher power intensity of the employed ultrasound, i.e., 690.34 kW/m^2^ as compared to lower power intensity of 20.38 kW/m^2^ used in Li et al. [60]. The high-power intensity might have broken down the SPW into smaller particles and enabled impurities other than starch granules to be purged during washing.

Table 1 shows that ultrasound-extracted starch had lower protein (0.1 ± 0.0% vs. 0.47 ± 0.1%) (*p* < 0.05) and ash contents (0.7 ± 0.1% vs. 0.9 ± 0.0%) (*p* < 0.05) than the control starch, while fat content was not detected in either starches. Lower protein content in the ultrasound-extracted starch could be due to the bond disruptions between protein and starch by ultrasound which eased the starch-protein separation [62]. Park et al. [63] and Zhang et al. [62] also obtained lower protein content of sorghum and corn starches in ultrasound-assisted extraction. Lower ash content is favourable for bioplastic film formation as higher ash content indicates higher mineral content which would hamper the formation of bioplastic film due to the possible interaction between the minerals and amylose, amylopectin and plasticizer [26]. In the present study, ultrasound-extracted starch exhibited lower protein and ash contents which had confirmed its higher starch purity than the control starch extracted by the conventional method.

### 3.6. Comparison of Optimum Reaction Conditions with Existing Literatures

The comparison of the optimum reaction conditions between this study and the reported literatures is presented in Table 2. The extraction yield of starch extracted from waste of sago pith in the present work was comparable with that of Pinyo et al. [20] using sago pith. It is worth to mention that even by utilizing the waste, it was still slightly more energy efficient as the ultrasonic amplitude and sonication duration required in the present study were lower (70 vs. 80%) and shorter (5 vs. 7 min). Moreover, with the extraction yield of more than 71.4%, SPW outperformed other feedstocks such as yam tuber, jicama roots and taro with lower extraction yields ranging from 19.0–32.1%. The low extraction yields reported could be due to the high solid loading used in jicama [16] starch suspension of ~25 wt.%, taro [8] and yam [14] starch suspensions of 50 wt.% which were 2.5 and 5 times higher than that in the present study (10 wt.%), respectively. As previously mentioned in Section 3.2, high solid loading could negatively affect mass transfer and cause a reduction in extraction yield. Apart from the high solid loading, lower duty cycle could be another contributing factor of low extraction yield for taro starch. Although the duty cycle in the present study was 1.7 times greater (83.3 vs. 50%), the extraction yield was 3.8 times higher (71.4 vs. 19.0%) with 2 times shorter sonication duration (5 vs. 10 min). This suggested that a suitable duty cycle is imperative as a high duty cycle would give rise to excessive heating and unnecessary electrical consumption, thus incurring more operational time and cost. Despite the influences of particle size and duty cycle towards extraction yield, they have not been investigated thoroughly in starch extraction thus far. Therefore, a more comprehensive investigation was conducted in the present study to elucidate their effects.

### 3.7. Characterization of Extracted Starches

#### 3.7.1. Surface Morphology

**Figure 5:**
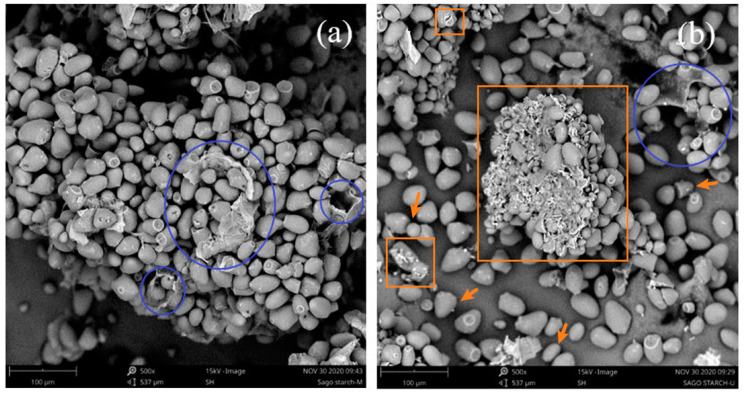
SEM micrographs of (**a**) control sago starch and (**b**) ultrasound-extracted sago starch at magnification of 500×. The cells that housed the starch granules were indicated by blue circle shapes; cracks and fissures were indicated by orange square shapes; structures of starch granules were indicated by orange arrows.

SEM images of the control starch extracted conventionally and the ultrasound-extracted starches are displayed in Figure 5a,b, respectively. As can be observed from the figure, particle agglomeration was found in both starches. The control starch had larger agglomerates which may be due to the presence of more residual protein content rendering the starch to be more viscous and allowing particles to attach with each other [64]. The residual protein content was evident from the cells that housed the starch granules as indicated by the blue circle shapes in Figure 5 [32]. From Table 1, the control starch was found to have higher protein content than the ultrasound-extracted starch (0.47 ± 0.25 vs. 0.1 ± 0.0%) which further corroborated the observation found from the SEM images. The granules of the control starch were oval with a temple bell-like shape and exhibited relatively smooth and intact surfaces, whereas in the ultrasound-extracted starch, the granules were of heterogeneous structures including oval, round, trigonal and oval with temple bell-like shapes. The average diameter of ultrasound-extracted starch was roughly 7% smaller than that of the control starch (34.1 ± 7.1 µm vs. 36.7 ± 7.4 µm). The particle size reduction after subjected to the cavitation effect of ultrasound was consistent with the studies reported by other researchers [65,66]. Incorporating smaller particle size in the preparation of bioplastic film would improve their distribution in the solution and thus producing a film with a more uniform matrix [67].

After ultrasound-assisted extraction, the starch granules exhibited uneven, disrupted and irregular surfaces with cracks and fissures as indicated by the orange square shapes in Figure 5b. It could be deduced that high-speed jets and violent shock waves produced from the collapsing bubbles had caused surface erosion and particle fragmentation while the starch surface was struck by the shock waves creating microfractures and crevices. This enabled more channels for water diffusion into the starch granules to facilitate the release of more starch contents during the extraction process [68]. This also explained the higher extraction yield obtained from ultrasound-assisted extraction than conventional extraction. Furthermore, ultrasound-extracted starch demonstrated better water absorption capacity than the control starch as granules breakdown by ultrasound can promote the water penetration into the starch granule [69]. Water absorption capacity is closely related to the swelling power and solubility which would further be discussed in Section 3.7.3.

#### 3.7.2. Particle Size Distribution

**Figure 6:**
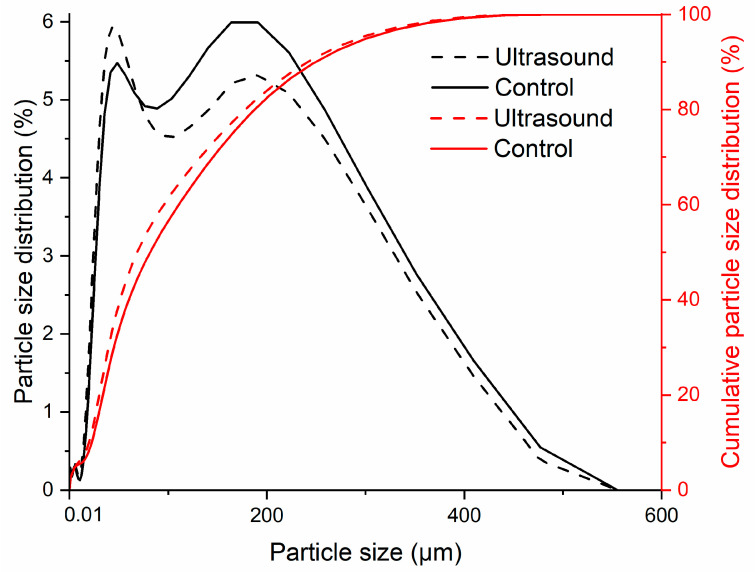
Particle size distribution and particle size distribution cumulative curves of control and ultrasound-extracted sago starches.

The particle size distribution and the cumulative particle size distribution curves of control and ultrasound-extracted starches that expressed in volume basis are presented in Figure 6. From the cumulative particle size distribution curves, it is noticed that both curves had reached the plateau for particle size larger than 477 µm. The particle size could be divided into two populations based on the number of peaks present in the particle size distribution curves. One of the populations was ranged from 10.5–88.9 µm and the other ranged from 103.6–477 µm. The control starch had equal size distribution of 47.3% for both the populations. Meanwhile, ultrasound-extracted starch had 51.9% size distribution of 10.5–88.9 µm and the remaining comprised of particle size ranged from 103.6–477 µm. It is important to take note that the particle size distribution data represented the size of isolated particle, minor aggregate and/or agglomerate forms [70] which supported the scenario of particle agglomeration as observed in SEM images.

#### 3.7.3. XRD

**Figure 7:**
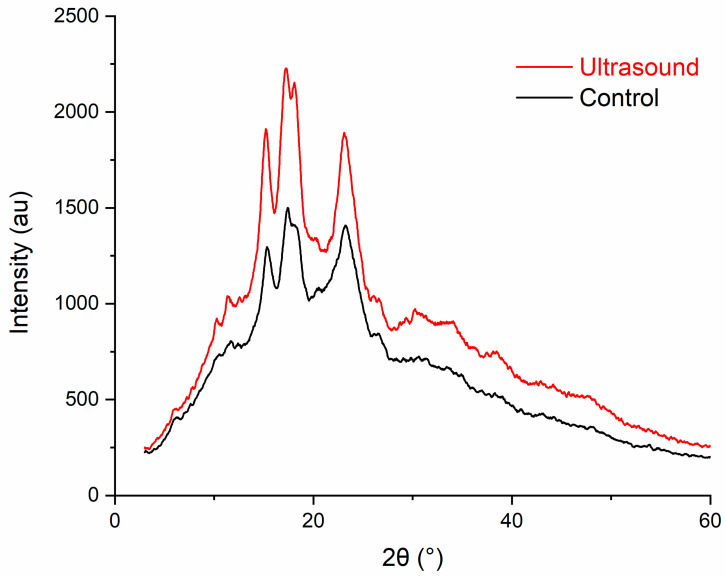
XRD patterns of control and ultrasound-extracted sago starches.

The XRD patterns of control and ultrasound-extracted starches are displayed in Figure 7. It was observed that sago starch depicted characteristic peaks of C-type starch at 2θ (5.90, 15.14, 17.05, 18.10 and 23°). The results were consistent with the results reported by Polnaya et al. [71] and Uthumporn et al. [72] on sago starch in which C-type patterns were also observed. It is evident that the diffraction pattern of ultrasound-extracted starch was similar to that of the control starch indicating that the crystalline structure of the starch remained unaltered after ultrasonication. The noticeable difference between the ultrasound-extracted and control starches was a slight reduction in the intensities of all the diffraction peaks after conventional extraction. The control starch had a slightly lower crystallinity of 2.1% as compared to the ultrasound-extracted starch (19.9 vs. 22.0%). The results obtained in the present study were different from those reported by Li et al. [60] and Zhu et al. [68]. The reason of control starch having lower crystallinity than ultrasound-extracted starch could be attributed to longer conventional extraction time of 30 min as compared with 5 min ultrasound extraction. Longer extraction duration promoted the swelling of the starch granule and more amylose could be leached out [61]. Since lower crystallinity would indicate higher amylose content, the amylose contents of ultrasound-extracted and control starches were then determined from Table 1. The latter (37.4 ± 0.0%) was found to have higher amylose content than the former (32.1 ± 0.0%) which was consistent with the crystallinity results.

#### 3.7.4. Swelling Power and Solubility

Amylose content is also associated with swelling power and solubility of starch in which lower amylose content would have higher swelling power and solubility [15]. The ultrasound-extracted starch exhibited higher swelling power (14.5 ± 0.0 vs. 11.8 ± 0.0 g gel/g starch) and solubility (29.3 ± 0.0% vs. 21.7 ± 0.1%) than the control starch. This was caused by the cavitation effects of ultrasound that disrupted the covalent bonds of crystalline structure and chains of sago starch as previously discussed. Therefore, more water molecules could bind to the free hydroxyl groups of amylose and amylopectin by hydrogen bonds to increase the swelling power and solubility of starch [69]. High swelling power and solubility of starch would be beneficial in bioplastic film fabrication as they could facilitate starch gelatinization and gel formation and thus obtain a more stable film-forming solution.

### 3.8. Characterization of Fabricated Bioplastic Films

The colour, mechanical and barrier properties of the bioplastic films prepared using control and ultrasound-extracted starches were evaluated in Table 3.

#### 3.8.1. Colour Properties

**Figure 8:**
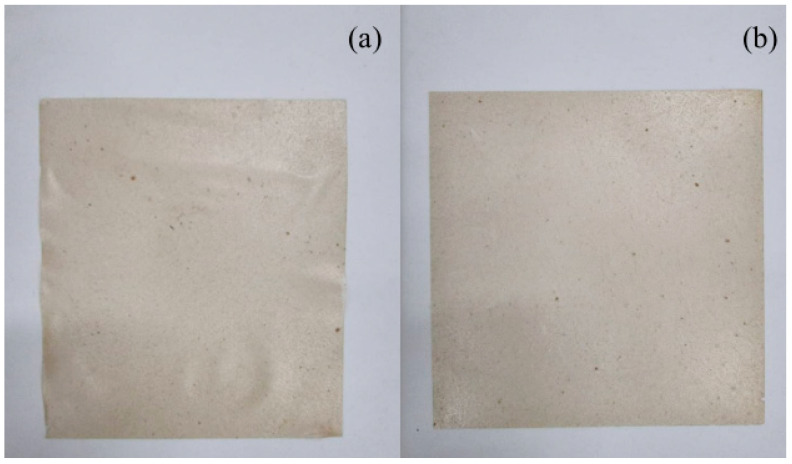
Photographs of film fabricated using (**a**) control starch and (**b**) ultrasound-extracted starch.

The photographs of bioplastic films fabricated using control and ultrasound-extracted starches are shown in Figure 8a,b, respectively. Both films were slightly brownish without any visible cracks. The surface exposed to air was rough whereas the surface in contact with the container was smooth and glossy. From Table 3, the film fabricated using ultrasound-extracted starch was found to have significant colour difference (ΔE) with lower *L* and *b* values than that using control starch (*p* < 0.05). These results indicated that the film fabricated using ultrasound-extracted starch was darker and less yellowish. This might be caused by the presence of impurities along with the extracted starch after ultrasonication [36].

#### 3.8.2. Mechanical Properties

Both films prepared using control and ultrasound-extracted starches had comparable mechanical properties (*p* > 0.05) as shown in Table 3. For film with ultrasound-extracted starch, tensile strength of 0.9 ± 0.1 MPa, Young’s modulus of 22.0 ± 0.8 MPa and elongation at break of 13.6 ± 2.0% were attained at glycerol loading of 40 wt.%. In the preparation of corn starch bioplastic film by Liu et al. [73], tensile strength of 1.3 ± 0.0 MPa, Young’s modulus of 1.8 ± 0.2 MPa and elongation at break of 14.0 ± 0.4% at glycerol loading of 10 wt.% were recorded. Lower tensile strength in the present study could be due to thinner film (0.11 vs. 0.19 mm) as compared to their study. In brief, the tensile test results revealed that sago starch with starch content of 87.8 ± 0.1% was sufficient and feasible to produce a bioplastic film with acceptable mechanical strength.

#### 3.8.3. Water Vapour Permeability (WVP)

Both the bioplastic films fabricated using control and ultrasound-extracted starches had comparable WVP of 1.13 ± 0.4 and 1.11 ± 0.1 × 10^−8^ g m^−1^ s^−1^ Pa^−1^ (*p* > 0.05), respectively. Slightly lower WVP of film fabricated using ultrasound-extracted starch could be attributed to smaller particle size of starch in producing a film with a uniform matrix. This would then reduce the interstitial spaces between the polymer structure and the diffusion rate of water molecules [74]. In the fabrication of corn starch bioplastic film by Ren et al. [38], the permeability of film was 7.89 × 10^−10^ g m^−1^ s^−1^ Pa^−1^ which was lower than that of both films in the present study. This could be due to higher glycerol loading used in the present study (40 vs. 20 wt.%) as compared to their study. Addition of glycerol which is more hydrophilic would increase the number of free hydroxyl groups and enhance the interaction with water molecules, making the film more favourable to adsorption and desorption of water molecules, thus increasing the WVP of films [75].

## 4. Conclusions

Ultrasound is a feasible method to extract starch from sago pith waste (SPW). It is not only capable of eliminating mass transfer limitations but also eradicating the needs of separate heating and agitation due to the localized temperature increment and the formation of micro jets. The extraction technique outshines conventional extraction with 17.2% higher extraction yield and much shorter sonication duration. Combination of ultrasound extraction and utilization of low-cost SPW rendered the process to be economically feasible and potentially sustainable for production of bioplastic film.

From the tensile and WVP tests of the bioplastic film fabricated using starch extracted from SPW, tensile strength of 0.9 MPa and WVP of 1.11 × 10^−8^ g m^−1^ s^−1^ Pa^−1^ were attained which had further confirmed that SPW could serve as an alternative starch source in the bioplastic film industries. Starch extracted from SPW would generate added-value product and close its life cycle and encourage circular economy.

Further improvement in the mechanical properties and WVP of bioplastic film could be achieved by incorporating different types of plasticizer or additives. Future studies could also be directed towards the investigation of factors such as ultrasonic tip placement, size and shape of the sono-reactor that would affect the efficiency of ultrasound, for instance, intensity and energy density. In addition, kinetic modelling of the ultrasound-assisted extraction process could be conducted to determine the proportion of acoustic energy transferred to the medium and thus designing a more efficient process. Furthermore, the possible starch gelatinization effect for large-scale ultrasonication extraction system also requires more studies in the future. This information would be beneficial for scaling the process to the industrial level.

## Figures and Tables

**Table 1:** Comparison between conventional and ultrasound-assisted extraction.

Parameters	Ultrasound	Conventional (Magnetic Stirring)
Extraction yield (%)	71.4 ± 0.4 ^a^	60.9 ± 1.2 ^b^
Extraction efficiency (%)	89.7 ± 0.5 ^a^	77.8 ± 1.5 ^b^
Reaction time (min)	5 ^a^	30 ^b^
Amylose content (%)	32.1 ± 0.0 ^a^	37.4 ± 0.0 ^b^
Starch content (%)	87.8 ± 0.1 ^a^	89.3 ± 0.1 ^b^
Protein content (%)	0.1 ± 0.0 ^a^	0.47 ± 0.1 ^b^
Ash content (%)	0.7 ± 0.1 ^a^	0.9 ± 0.0 ^b^
Fat content (%)	n.d.	n.d.

Means ± standard deviation in the same row with different superscripts are significantly different (*p* < 0.05). n.d denotes for not detected.

**Table 2:** Comparison of optimum reaction conditions for ultrasound-assisted starch extraction in various studies.

Feedstock	Particle Size (µm)	Solid Loading (wt.%)	Sonication Duration (min)	Ultrasonic Amplitude (%)	Duty Cycle (%)	Extraction Yield (%)	Purity (%)	Ref.
SPW	Smaller than 250	10	5	70	83.3	71.4	87.8	Present study
Sago pith	-	10	7	80	-	71.5	95.0	[20]
Yam tuber	-	50	15 ^a^	70 ^a^	-	32.1	-	[14]
Jicama roots	-	25	10 ^a^	-	-	24.0	80.9	[16]
Taro	-	50	10 ^a^	50 ^a^	50 ^a^	19.0 *	-	[8]

* amount of pure starch. ^a^ Parameters investigated.

**Table 3:** Properties of film fabricated using control and ultrasound-extracted starches.

	Film Fabricated Using	Control Starch	Ultrasound-Extracted Starch
Colour properties	L	84.4 ± 0.4 ^a^	80.9 ± 0.0 ^b^
	a	1.14 ± 0.0 ^a^	1.13 ± 0.0 ^a^
	b	12.1 ± 0.1 ^a^	11.9 ± 0.0 ^b^
	ΔE	13.7 ± 0.0 ^a^	15.6 ± 0.1 ^b^
Mechanical properties	Tensile strength (MPa)	0.9 ± 0.3 ^a^	0.9 ± 0.1 ^a^
	Young’s Modulus (MPa)	22.6 ± 1.1 ^a^	22.0 ± 0.8 ^a^
	Elongation at break (%)	13.8 ± 1.8 ^a^	13.6 ± 2.0 ^a^
Barrier property	WVP × 10^−8^ (g m^−1^ s^−1^ Pa^−1^)	1.13 ± 0.4 ^a^	1.11 ± 0.1 ^a^

Means ± standard deviation in the same row with different superscripts are significantly different (*p* < 0.05).
